# Effects of the kinetic pattern of dietary glucose release on nitrogen utilization, the portal amino acid profile, and nutrient transporter expression in intestinal enterocytes in piglets

**DOI:** 10.1186/s40104-024-01000-z

**Published:** 2024-03-18

**Authors:** Zexi Li, Yunfei Li, Yufei Zhao, Guifu Wang, Rujie Liu, Yue Li, Qamar Aftab, Zewei Sun, Qingzhen Zhong

**Affiliations:** 1 Jilin Province Key Laboratory of Animal Nutrition and Feed Science, College of Animal Science and Technology, Key Lab of Animal Production, Product Quality and Security, Ministry of Education, Jilin Agricultural University, 2888 Xincheng Street, Changchun City, Jilin Province People’s Republic of China; 2 Dongfeng County Sika Deer Industry Development Service Center, Dongfeng County, Liaoyuan City, Jilin Province China

**Keywords:** Glucose release kinetics, Nitrogen utilization, Nutrient transporter, Piglet

## Abstract

**Background:**

Promoting the synchronization of glucose and amino acid release in the digestive tract of pigs could effectively improve dietary nitrogen utilization. The rational allocation of dietary starch sources and the exploration of appropriate dietary glucose release kinetics may promote the dynamic balance of dietary glucose and amino acid supplies. However, research on the effects of diets with different glucose release kinetic profiles on amino acid absorption and portal amino acid appearance in piglets is limited. This study aimed to investigate the effects of the kinetic pattern of dietary glucose release on nitrogen utilization, the portal amino acid profile, and nutrient transporter expression in intestinal enterocytes in piglets.

**Methods:**

Sixty-four barrows (15.00 ± 1.12 kg) were randomly allotted to 4 groups and fed diets formulated with starch from corn, corn/barley, corn/sorghum, or corn/cassava combinations (diets were coded A, B, C, or D respectively). Protein retention, the concentrations of portal amino acid and glucose, and the relative expression of amino acid and glucose transporter mRNAs were investigated. In vitro digestion was used to compare the dietary glucose release profiles.

**Results:**

Four piglet diets with different glucose release kinetics were constructed by adjusting starch sources. The in vivo appearance dynamics of portal glucose were consistent with those of in vitro dietary glucose release kinetics. Total nitrogen excretion was reduced in the piglets in group B, while apparent nitrogen digestibility and nitrogen retention increased (*P* < 0.05). Regardless of the time (2 h or 4 h after morning feeding), the portal total free amino acids content and contents of some individual amino acids (Thr, Glu, Gly, Ala, and Ile) of the piglets in group B were significantly higher than those in groups A, C, and D (*P* < 0.05). Cluster analysis showed that different glucose release kinetic patterns resulted in different portal amino acid patterns in piglets, which decreased gradually with the extension of feeding time. The portal His/Phe, Pro/Glu, Leu/Val, Lys/Met, Tyr/Ile and Ala/Gly appeared higher similarity among the diet treatments. In the anterior jejunum, the glucose transporter *SGLT1* was significantly positively correlated with the amino acid transporters *B*^*0*^*AT1*, *EAAC1*, and *CAT1*.

**Conclusions:**

Rational allocation of starch resources could regulate dietary glucose release kinetics. In the present study, group B (corn/barley) diet exhibited a better glucose release kinetic pattern than the other groups, which could affect the portal amino acid contents and patterns by regulating the expression of amino acid transporters in the small intestine, thereby promoting nitrogen deposition in the body, and improving the utilization efficiency of dietary nitrogen.

## Background

Energy and protein are widely recognized as the two primary components of pig diets [1, 2]. Starch, which is found primarily in cereals, is the main energy source used in modern intensive pig production [3]. Upon enzymatic hydrolysis in the pig intestine, glucose from starch is subsequently absorbed and provides direct energy to the animal [4]. The various botanic origins and different physicochemical properties of starch may cause variations in the starch digestion rate and glucose release kinetics in the gastrointestinal tract [5], thus impacting piglet growth performance and diet nitrogen utilization, the extent of which dependent on glucose release kinetic from starch [6]. Intestinal mucosal energy metabolism is very complex because the energy source for the intestinal mucosa is a complex mixture of arterial and luminal substrates, and the pattern of intestinal substrate oxidation can be altered by the nutrient composition in the diet [7]. Stoll et al. [8] showed that enteral glucose, arterial glutamine, arterial glucose, and enteral glutamate contributed 15%, 19%, 29% and 36%, respectively, to the total production of CO_2_ in the portal drained viscera (PDV) of piglets. Moreover, approximately three-fourths of the energy requirement of the PDV was provided by the oxidation of glucose, glutamine, and glutamate. When glucose is insufficient or lags the supply of amino acids, amino acids will oxidize to provide energy to the small intestinal cell under fuel-shortage conditions [9–11]. A timely supply of glucose can minimize the oxidation of absorbable amino acids in the digestive tract, as intestinal cells utilize glucose for energy supply, thereby enhancing the likelihood and efficiency of dietary protein deposition into body protein. Consequently, the rate and extent of dietary glucose release in different parts of the intestine are closely related to nitrogen deposition in pigs [12].

In recent years, an increasing number of studies have noted that promoting the synchronization of glucose and amino acid release in the digestive tract of animals can effectively improve dietary nitrogen utilization [13]. Zhou et al. [6] used different purified starches to regulate dietary glucose release kinetics in growing pigs, further confirming that an appropriate dietary glucose release profile may effectively strengthen protein deposition in the body and improve protein utilization efficiency and growth performance in pigs.

Although in vitro starch digestion kinetics, and their effects on animal performance and nitrogen utilization have been studied extensively [6, 14–16], there are limited studies on the effects of diet with different glucose release profiles on the portal amino acid concentration and profile, and on the expression of glucose and amino acid transporters in piglets. Studies on this subject are therefore needed.

In the present study, we hypothesized that a diet with an optimal glucose release kinetic pattern can improve the utilization efficiency of dietary nitrogen by changing the serum amino acid content and composition in the portal vein, and these changes may be associated with the expression levels of genes involved in glucose and amino acid transport in piglets. Therefore, this study aimed to investigate the effects of dietary glucose release kinetics on nitrogen utilization, hepatic portal amino acid composition, and the expression of nutrient transporter genes in the intestinal mucosa in piglets.

## Materials and methods

### Animals, experimental design, and diets

Sixty-four Duroc × Landrace × Yorkshire weaned barrows, with an average body weight of 15.00 ± 1.12 kg, were obtained from the Changchun Aoxin Agriculture and Animal Husbandry Development Co., Ltd. (China), and were randomly allocated to a completely randomized design with 4 dietary treatment groups. The experimental diets were formulated with different starch sources: corn (group A), corn/barley (group B), corn/sorghum (group C), and corn/cassava (group D) combinations. All the experimental diets were formulated to contain similar levels of crude protein (CP), net energy (NE), and starch to meet the nutrient requirements of the National Research Council [17] (Tables 1 and 2). The feeding trial continued for 5 weeks, including a 7-d acclimation period and a 28-d experimental period. All the piglets in the study were housed individually in stainless steel metabolic cages at the experimental site of Jilin Agricultural University. The daily feed was divided into three equal meals which were given to each pig at 07:00, 12:00, and 17:00. All the pigs had access to fresh water ad libitum. The temperature was maintained at 23 ± 2 °C. The humidity varied from 55% to 65% during the experiment. On d 7, 14, 21 and 28, feed intake and body weight were recorded, and peripheral blood was sampled on 28 d. The growth performance and peripheral blood parameter characteristics have been reported previously [18].

**Table 1:** Analyzed composition of ingredients (as-fed basis)

Item	Corn	Barley	Sorghum	Cassava	Soybean meal	Whey	Fish meal	Limestone	Dicalcium phosphate
Dry matter, %	85.50	87.25	88.77	87.08	86.35	99.09	92.78	99.62	98.00
Crude protein, %	7.43	10.69	8.76	2.96	43.65	3.67	68.21	-	-
Ether extract, %	3.16	1.68	2.96	0.70	1.25	0.73	8.57	-	-
Ash, %	1.18	2.13	1.03	1.84	5.66	7.86	17.43	98.00	98.00
NDF, %	7.85	15.94	6.01	11.56	12.33	-	-	-	-
ADF, %	2.09	5.35	1.00	2.25	7.41	-	-	-	-
Ca, %	0.02	0.07	0.02	0.09	0.33	0.82	3.33	38.00	21.90
P, %	0.24	0.33	0.20	0.08	0.68	0.73	2.69	0.01	16.00

**Table 2:** Ingredients and nutrient composition of experimental diets (as-fed basis)

Items	A (corn)	B (corn/barley)	C (corn/sorghum)	D (corn/cassava)
Ingredient, %				
Corn	63.76	52.90	51.31	51.39
Barley	-	10.91	-	-
Sorghum	-	-	11.90	-
Cassava	-	-	-	10.82
Soybean meal	18.80	19.80	19.62	20.93
Whey	4.60	3.80	4.52	3.50
Fish meal	5.50	5.20	5.00	6.20
Soybean oil	4.40	4.50	4.65	4.50
L-Lysine-HCl (78%)	0.28	0.25	0.29	0.20
DL-Methionine (99%)	0.13	0.11	0.13	0.12
L-Threonine	0.05	0.04	0.05	0.03
Tryptophan (100%)	0.03	0.03	0.03	0.02
Dicalcium phosphate	0.35	0.36	0.40	0.24
Limestone	0.80	0.80	0.80	0.75
Salt	0.30	0.30	0.30	0.30
Vitamin and mineral premix^a^	1	1	1	1
Calculated composition				
Net energy, MJ/kg^b^	10.94	10.89	10.92	10.87
Crude protein, %	17.30	17.82	17.45	17.98
Ash, %	5.38	5.43	5.36	5.43
Neutral detergent fiber, %	7.32	8.33	7.16	7.86
Acid detergent fiber, %	2.73	3.16	2.65	2.87
Calcium, %	0.68	0.67	0.67	0.66
Phosphorus, %	0.52	0.52	0.51	0.51
Standardized ileal digestible amino acids, %^c^				
Lysine	1.14	1.14	1.14	1.14
Methionine + Cysteine	0.62	0.62	0.62	0.62
Threonine	0.40	0.39	0.40	0.40
Tryptophan	0.61	0.61	0.61	0.61
Analyzed composition				
Dry matter, %^d^	87.69	87.77	88.09	87.76
GE, MJ/kg^d^	17.18	17.15	17.16	17.17
Total starch, %	44.53	44.26	44.77	44.48
Amylose/amylopectin	0.44	0.38	0.23	0.41

^a^Premix provided the following per kg of complete diet for piglets: 28,500 IU vitamin A, 6,000 IU vitamin D_3_, 67.5 IU vitamin E, 7.5 mg vitamin K_3_, 7.5 mg vitamin B_1_, 15 mg vitamin B_2_, 9 mg vitamin B_6_, 0.075 mg vitamin B_12_, 70 mg niacin, 3 mg folacin, 37.5 mg pantothenic calcium, 105 mg choline, 0.375 mg biotin, 0.15 mg antioxidants, 1 mg Co, 145 mg Cu, 155 mg Fe, 75 mg Mn, 125 mg Zn, 0.3 mg I, 0.3 mg Se

^b,c^Values were estimated based on a database of NRC [17]

^d^Analytical results were obtained according to AOAC [19]

### Simulation of glucose release processes in vitro

The methods described by Englyst et al. [20] and Hung and Morita [21] were used to determine the in vitro digestion of dietary starch, with light modifications. Briefly, samples (approximately 1 g, 1 mm screen) were incubated in a 10 mL pepsin solution (pH 2.0) that contained 0.05 g pepsin (P-7000; Sigma Aldrich, Darmstadt, Germany) and 0.05 g guar gum (P-9000-30-0; Sigma Aldrich, Darmstadt, Germany) in 0.05 mol/L HCl at 37 °C for 30 min with vibration. Then infunde into 10 mL of 0.25 mol/L sodium acetate (C_2_H_3_NaO_2_) solution and 5 mL of an enzyme mixture with 0.7 g pancreatin (Sigma P-7545; Sigma Aldrich, Darmstadt, Germany), 0.05 mL amyloglucosidase (A-7095; Sigma Aldrich, Milan, Italy), and 3 mg invertase (Sigma I-4504; Sigma Aldrich, Darmstadt, Germany); the pH of the solution is 6.86. Incubations were carried out at 39 °C under horizontal agitation. After incubating for 0, 15, 30, 60, 90, 120, 180, or 240 min, an aliquot of 0.5 mL was taken, respectively, to which absolute ethanol was added to stop the digestion of the starch. Then the samples were subsequently centrifuged at 3,000 × *g* for 10 min to obtain the supernatant. The glucose content of the supernatant was measured using a glucose oxidase kit (Megazyme, Bray, Ireland).

$$\mathrm{Glucose\ release},\mathrm{ \%}/\mathrm{min }=\left(\Delta t/\left(t^{\prime}- t\right)/TS\right)\times 100\mathrm{\%}$$

where *t'* is the latter of the adjacent time points, *t* is the former of the adjacent time points, *∆t* is the difference in the content of glucose release content between *t* and *t'*, and *TS* is the total starch in the diet.

### Nitrogen balance experiment

Feces and urine were collected from each pig from 30 to 34 d. Feces were collected in self-sealing bags, and urine was collected in wide-mouth bottles with stoppers. After collection, the feces, and urine were treated with 10% sulfuric acid for nitrogen fixation and stored in a low-temperature refrigerator at −20 °C for nitrogen determination.

Nitrogen concentrations in the diet, feces, and urine were measured by an automatic Kjeldahl nitrogen determination apparatus (Kjeltec 8420, FOSS Analytical Co., Beijing, China). The main formulae are as follows:

$$\mathrm{Nitrogen\ intake }\ ({\text{NI}},\mathrm{ g}/{\text{d}})\hspace{0.17em}=\hspace{0.17em}\mathrm{average\ daily\ feed\ intake}\hspace{0.17em}\times \hspace{0.17em}\mathrm{feed\ nitrogen\ content}$$

$$\mathrm{Fecal\ nitrogen }\ ({\text{FN}},\mathrm{ g}/{\text{d}})\hspace{0.17em}=\hspace{0.17em}\mathrm{average\ daily\ fecal\ weight}\hspace{0.17em}\times \hspace{0.17em}\mathrm{fecal\ nitrogen\ content}$$

$$\mathrm{Urinary\ nitrogen }\ ({\text{UN}},\mathrm{ g}/{\text{d}})\hspace{0.17em}=\hspace{0.17em}\mathrm{average\ daily\ urine\ volume}\hspace{0.17em}\times \hspace{0.17em}\mathrm{urinary\ nitrogen\ content}$$

$$\mathrm{Absorbed\ N }\ ({\mathrm {AN}},\mathrm{ g}/{\mathrm {d}}) =\mathrm{ NI }-\hspace{0.17em}{\mathrm{FN}}$$

$$\mathrm{Nitrogen\ retention }\ ({\text{NR}},\mathrm{ g}/{\text{d}})\hspace{0.17em}=\hspace{0.17em}{\text{NI}}\hspace{0.17em}-\hspace{0.17em}{\text{FN}}\hspace{0.17em}-\hspace{0.17em}{\text{UN}}$$

$$\mathrm{Apparent\ nitrogen\ digestibility }\ ({\text{ND}},\mathrm{ \%}) =\mathrm{ AN }\times 100 /{\text{NI}}$$

$$\mathrm{Biological\ value\ of\ feed\ protein }\ ({\text{BVFP}}) =\mathrm{ NR }\times 100/{\text{AN}}$$

### Portal glucose and amino acid analysis

On d 35, after 2 h and 4 h of the morning and afternoon feeding, four piglets per group were anesthetized by rapid intraperitoneal injection of a 4% solution of sodium pentobarbital, which was prepared according to their body weight (1.2 mL/kg). Blood (20 mL) was collected from the portal vein. Serum-free amino acids were determined using an automated amino acid analyzer (Membrapure GmbH A300, Berlin, Germany), and serum glucose was determined using a glucose oxidase kit (Megazyme, Bray, Ireland).

### Expression of glucose and amino acid transporter mRNAs

The middle sections (4–5 cm) of the duodenum, anterior jejunum, posterior jejunum, and ileum were sampled, and the portal vein blood was collected. Then, the samples were rinsed with saline water at 4 °C, and the surface water was blotted off with filter paper. The mucosa of duodenum, anterior jejunum, posterior jejunum, and ileum were scraped on autoclaved slides, placed in 1.5 mL EP tubes, frozen in liquid nitrogen and subsequently transferred to a −80 °C refrigerator for relative quantification of the sodium-glucose cotransporter-1 (*SGLT1*), neutral amino acid transporter *B*^*0*^*AT1*, excitatory amino acid carrier 1 (*EAAC1*), and cationic amino acid transporter 1 (*CAT1*) genes using the staining method.

Total RNA was extracted from the frozen intestinal mucosa samples using TRizol reagent (Takara Biotechnology, Dalian, China) according to the manufacturer’s instructions. The RNA quality and concentration of each sample were measured using a Nanodrop-1000 spectrophotometer (Thermo Fisher Scientific Inc., Waltham, MA, USA), and the ratio (OD_260_:OD_280_) ranged from 1.8 to 2.0. Thereafter, 1 μg of total RNA was used to produce cDNA using a synthesis kit (Takara Biotechnology, Dalian, China) according to the manufacturer’s instructions. Real-time PCR of the *β-actin*, *SGLT1*, *B*^*0*^*AT1*, *EAAC1 *and *CAT1* genes were performed on a CFX96 Real-time PCR Detection System (Bio-Rad Laboratories, Hercules, CA, USA) using TBGreen™ Premix Ex Taq™ (Takara Biotechnology, Dalian, China) in a total volume of 20 μL, after which the proteins were analyzed in triplicate. The specific sequences of primer used in this study were listed in Table 3. The protocols for all genes included a denaturation program (30 s at 95 °C), an amplification and quantification program repeated for 40 cycles (5 s at 95 °C, 30 s at 60 °C), followed by the melting curve program at 65–95 °C with a heating rate of 0.1 °C/s. The relative quantification of gene amplification by real-time PCR was determined using the value of the threshold cycle (Ct). To normalize the mRNA expression levels of each target gene, *β-actin* was used as the internal control, and the relative expression levels were calculated using the 2^−ΔΔCT^ method [22].

**Table 3:** Primers used for real-time PCR analysis

Gene	Primer sequence (5´→3´)	Length, bp	Annealing temperature, °C	Accession No.
*β-actin*	F:GGATGCAGAAGGAGATCACG	130	59.8	U07786.1
	R:ATCTGCTGGAAGGTGGACAG			
*SGLT1*	F:GGCTGGACGAAGTATGGTGT	153	58.8	M34044.1
	R:ACAACCACCCAAATCAGAGC			
*B0AT1*	F:ACAACAACTGCGAGAAGGAC	149	57.8	DQ231579.1
	R:GATAAGCGTCAGGATGTTCG			
*EAAC1*	F:AAGAGCCAGGTGGATAAGAG	118	55.8	JF521497.1
	R:CATTCAACTGTGCGATAAAC			
*CAT1*	F: TGCCCATACTTCCCGTCC	192	59.6	NM_001012613
	R: GGTCCAGGTTACCGTCAG			

### Statistical analysis

The IBM SPSS 19.0 statistics software package (IBM Corp., Armonk, NY, USA) was used to perform the data analysis. All the data were checked for a normal distribution and homogeneous variance using Levene’s test procedure, One-way analysis of variance (ANOVA) followed by Tukey’s post-hoc test was subsequently used to analyze all the experimental data. The Euclidean distance was calculated after the portal amino acid concentrations were log-standardized. Based on Euclidean distance, cluster analysis was performed to compare the differences in portal amino acid patterns of piglets among the different treatment groups. Correlation analysis was used to analyze the relationship between the mRNA expression of glucose and amino acid transporters in the intestinal epithelium. The cluster analysis loading heatmap and correlation analysis loading heatmap were generated with the Origin 2021 Pro software package (OriginLab, Northampton, MA, USA). The data were expressed as the mean and standard deviation. Differences at a *P*-value ≤ 0.01 were considered highly significant; differences at a *P*-value ≤ 0.05 were considered significant.

## Results

### Dietary starch source and composition affected in vitro glucose release kinetics

**Fig. 1:**
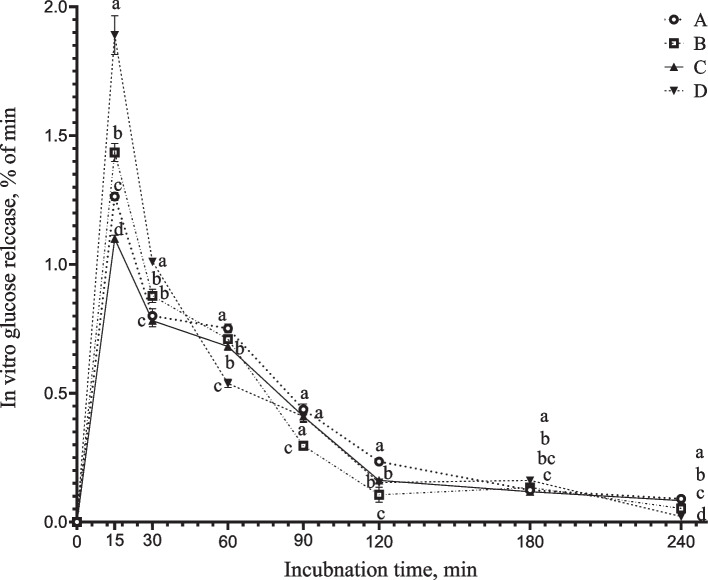
Dietary glucose releases in in vitro experiment. ^a–d^Different letters indicate statistically significant differences between the various diets at the same in vitro digestion time points (*P* < 0.05)

The four diets, each with different starch sources, exhibited variations in their glucose release kinetics (Fig. 1). After 15 min of in vitro digestion, all the diets reached their peak glucose release rate, and the difference among the four dietary was significant (*P* < 0.05). The corn/cassava diet in group D demonstrated the highest glucose release rate, followed by the corn/barley diet in group B, the corn alone in group A, and the corn/sorghum diet in group C (*P* < 0.05). After 15 min, all the diets exhibited varying degrees of decline in the glucose release rate. From 15 to 30 min, the corn/cassava diet had the greatest decrease in the glucose release rate, while the corn/sorghum diet had the least (*P* < 0.05). The linear slopes between these two points were –0.06 for group D, –0.04 for group B, –0.03 for group A and –0.02 for group C. At 30 min of in vitro digestion, there was no significant difference observed between groups A and B regarding their rate of glucose release as the decrease in glucose release rate was greater in group B than that observed in group A. From 30 to 60 min of in vitro digestion, the glucose release rate continued to decline sharply for corn/cassava diet followed by corn/barley diet, whereas for both the corn and corn/sorghum diets, the downward tended to be relatively flat, with interpoint slopes of –0.02, –0.006, –0.002 and –0.003, respectively. After 60 min of in vitro digestion, the lowest glucose release rate was observed for corn/cassava diet (*P* < 0.05), while the highest rate was observed for the corn/barley diet (*P* < 0.05) which closely resembled that observed for corn/sorghum diet. After 90 min, there was still variation in the decreasing trend of the glucose release rate among all dietary groups, with greater fluctuations observed in groups B and D than in groups A and C. Until 240 min of in vitro digestion, the glucose release rates remained significantly different among the four groups, with group A demonstrating the highest rate, followed by groups C, B, and D (*P* < 0.05).

### Nitrogen utilization was affected by diet glucose release kinetics

In the present study, all four experimental diets with different glucose release kinetics had no significant effect on nitrogen intake in piglets. However, compared with the other diet treatments, the diet B treatment significantly decreased fecal nitrogen, urinary nitrogen, and total nitrogen excretion (*P* < 0.05; Table 4) and markedly increased nitrogen retention and apparent digestibility (*P* < 0.05). Although the fecal nitrogen of pigs in groups C and D increased (*P* < 0.05), the urinary nitrogen decreased more significantly (*P* < 0.05), and the total nitrogen excretion of the piglets in group D was still lower than that in group A (*P* < 0.05). From the perspective of nitrogen retention and biological value of feed protein, the glucose release profile in both B and D diets can increase the nitrogen retention of piglets.

**Table 4:** Nitrogen balance of piglets in response to the diets with different glucose release kinetic profiles

Item	A	B	C	D	SEM	*P*-value
Nitrogen intake, g/d	39.29	39.58	39.70	40.01	0.19	0.57
Fecal nitrogen, g/d	6.27^b^	5.69^c^	7.26^a^	6.91^a^	0.17	< 0.001
Urinary nitrogen, g/d	7.38^a^	5.73^b^	6.04^b^	4.95^c^	0.25	< 0.001
Total nitrogen excretion, g/d	13.65^a^	11.42^b^	13.30^a^	11.86^b^	0.27	< 0.001
Absorbed nitrogen, g/d	33.02^ab^	33.52^a^	31.90^c^	32.70^b^	0.16	< 0.001
Nitrogen retention, g/d	25.64^b^	28.16^a^	26.40^b^	28.15^a^	0.33	< 0.001
Nitrogen apparent digestibility, %	84.04^b^	85.62^a^	81.71^c^	82.73^c^	0.41	< 0.001
Biological value of feed protein (BVFP)	77.66^b^	84.02^a^	82.78^a^	86.10^a^	0.94	< 0.001

^a–c^Means in the same row with different superscripts differ (*P* ≤ 0.05)

### Changes in portal glucose levels in piglets fed diets with different glucose release kinetics

**Fig. 2:**
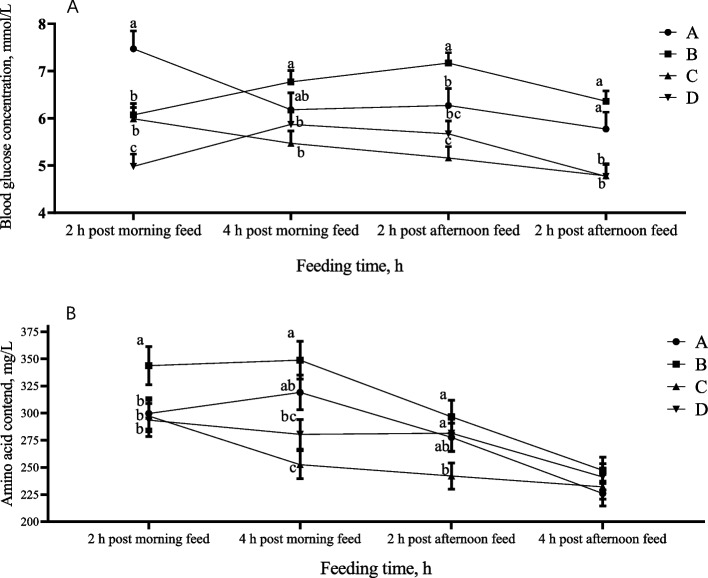
Variation of portal glucose and amino acids concentration of piglets after feeding. **A** Glucose. **B** Amino acid. ^a–c^Different letters indicate statistically significant differences among the four diets at the same time after feeding (*P* < 0.05)

The pigs exhibited distinct patterns in portal glucose levels following the consumption of diets with different glucose release kinetics (Fig. 2A). Group A piglets fed the corn diet showed a consistent decrease from the peak level at 2 h after the morning feeding. Group B piglets fed the corn/barley diet had an intermediate blood glucose concentration at 2 h after the morning feeding, followed by a significant increase that surpassed that of the other three groups (*P* < 0.05). Although there was no significant difference in blood glucose concentration between groups C and B at 2 h after the morning feeding (*P* > 0.05), and the level subsequently showed a decreasing trend thereafter. The piglets in group D exhibited the lowest blood glucose concentration at 2 h after the morning feeding, after which the blood glucose concentration exhibited a parabolic linear trend.

### Portal amino acid concentrations and patterns differ among piglets receiving different glucose release kinetic diet treatments

The kinetic patterns of dietary glucose release can also significantly impact the portal total free amino acid content of piglets, as illustrated in Fig. 2B. The absorption trend of total free amino acids gradually decreased from 2 h after the morning feeding to 4 h post afternoon feeding. At both 2 h and 4 h after the morning feeding, the portal concentration of total free amino acids in the piglets in group B was significantly higher than that in groups A, C, and D (*P* < 0.05). At 2 h after the afternoon feeding, the total free amino acid level in group B decreased to the level of groups A and C, and any significant differences among the piglets within the four diet treatment groups disappeared 4 h after afternoon feeding.

In this experiment, the portal concentrations of 14 test amino acids were significantly influenced by diet treatment and time after feeding, with a significant interaction effect observed between diet treatment and time after feeding (Table 5). Compared to those in group A, which received the traditional corn/soybean meal diet, the portal amino acid concentrations in group B significantly changed. Lys, Met, Ile, Phe, Gly, Tyr, Ala, and Pro were significantly more abundant in group B (*P* < 0.05), while Thr, Arg, and Glu were significantly less abundant (*P* < 0.05). Conversely, the Lys, Met, Thr, Leu, Val, Arg, and His concentrations in group C, and Leu, Ile, Arg, Glu, and Pro concentrations in group D were lower than those in group A (*P* < 0.05). Overall, the majority of test amino acids exhibited a decrease in portal concentration as morning and afternoon feeding times increased.

**Table 5:** The portal amino acids patterns of the piglets in different diet treatments after 2h and 4h of the morning feeding or afternoon feeding

Item	Treatment				SEM	Hour post-morning feeding		Hour post-afternoon feeding		SEM	*P*-value		
	A	B	C	D		2	4	2	4		diet	time	Diet × time
Lys	6.76^b^	7.88^a^	5.24^c^	6.34^b^	0.23	7.98^a^	5.85^c^	6.98^b^	5.41^c^	0.23	< 0.001	< 0.001	< 0.001
Met	6.17^ab^	6.31^a^	4.70^c^	5.39^b^	0.20	7.38^a^	6.59^b^	4.77^c^	3.85^d^	0.20	< 0.001	< 0.001	< 0.001
Thr	35.69^a^	33.86^ab^	32.49^b^	33.19^b^	0.48	34.71^ab^	33.36^bc^	34.88^a^	32.28^c^	0.48	0.001	0.002	< 0.001
Leu	17.18^b^	18.71^a^	15.97^c^	14.42^d^	0.38	17.54^a^	18.32^a^	16.35^b^	14.07^c^	0.38	< 0.001	< 0.001	< 0.001
Ile	8.88^bc^	11.35^a^	9.21^b^	8.42^c^	0.23	11.67^a^	10.60^b^	8.81^c^	6.79^d^	0.23	< 0.001	< 0.001	< 0.001
Val	15.27^bc^	18.48^a^	14.73^c^	16.23^b^	0.37	19.33^a^	17.93^b^	15.06^c^	12.39^d^	0.37	< 0.001	< 0.001	< 0.001
Phe	7.31^b^	8.18^a^	7.40^b^	6.65^b^	0.25	9.67^a^	8.29^b^	6.27^c^	5.33^d^	0.25	0.002	< 0.001	< 0.001
Arg	17.69^a^	16.70^a^	11.89^c^	13.37^b^	0.31	16.89^a^	13.86^c^	14.46^bc^	14.80^b^	0.31	< 0.001	< 0.001	< 0.001
His	7.48^b^	7.24^b^	5.96^c^	9.66^a^	0.27	8.99^a^	7.64^b^	6.88^b^	5.88^c^	0.27	< 0.001	< 0.001	< 0.001
Glu	22.40^a^	19.05^b^	22.36^a^	19.21^b^	0.47	25.43^a^	24.39^a^	20.48^b^	12.73^c^	0.47	< 0.001	< 0.001	< 0.001
Gly	53.17^b^	56.81^a^	53.55^b^	58.19^a^	0.61	53.62^b^	64.11^a^	52.89^b^	51.10^c^	0.61	< 0.001	< 0.001	< 0.001
Tyr	12.25^ab^	12.86^a^	12.49^a^	11.38^b^	0.13	15.47^a^	12.56^b^	10.91^c^	10.40^c^	0.30	0.009	< 0.001	< 0.001
Pro	24.71^b^	30.76^a^	25.02^b^	31.53^a^	0.45	29.78^a^	27.56^b^	26.77^b^	27.91^b^	0.45	< 0.001	0.001	< 0.001
Ala	44.92^ab^	45.26^a^	43.25^b^	42.64^b^	0.60	46.21^b^	50.59^a^	46.29^b^	32.96^c^	0.60	0.011	< 0.001	< 0.001

^a–d^Means in the same row with different superscripts differ (*P* ≤ 0.01)

**Fig. 3:**
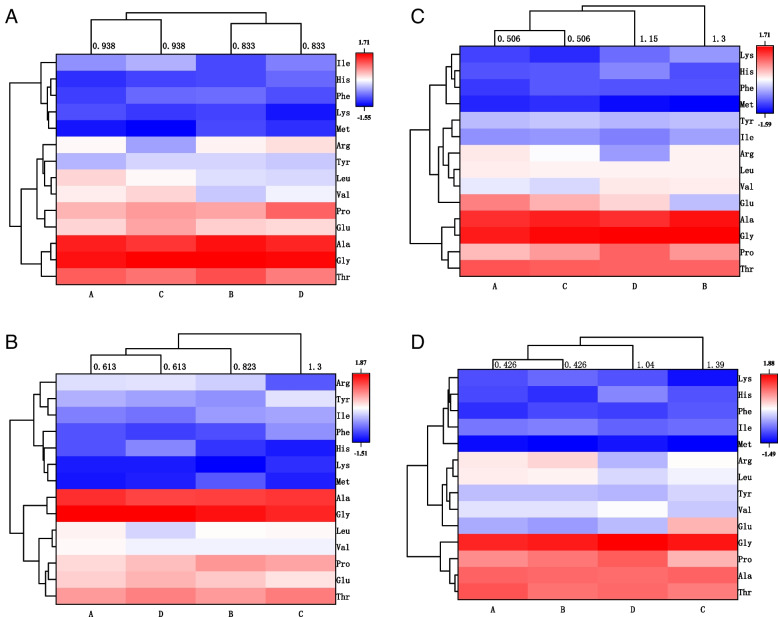
Cluster analysis loading heatmap showing similarity between amino acid profiles of different treatment groups at the different time points after feeding. **A** 2 h post morning feeding. **B** 4 h post morning feeding. **C** 2 h post afternoon feeding. **D** 4 h post afternoon feeding

The portal amino acid patterns of the piglets in each treatment group at different times after feeding are illustrated in Fig. 3. As the feeding time increased, the difference in portal amino acid patterns gradually decreased based on the Euclidean distance of cluster analysis. At 2 h after morning feeding, there were significant differences observed among the portal amino acid patterns of the piglets in all the groups. The amino acid patterns of group A and group C exhibited a certain degree of similarity when the Euclidean distance reached 0.833, while the amino acid patterns of group B and group D exhibited a similar extent of resemblance when the Euclidean distance reached 0.938; however, significant differences still existed between the A, C and B, D amino acid patterns. The changes in the portal concentrations of His/Phe, Lys/Met, Leu/Val, and Ala/Gly were relatively consistent across all the experimental treatments, particularly demonstrating the high consistency of Ala and Gly. At 4 h after the morning feeding, the amino acid pattern of group C differed significantly from that of the other three groups; however, there were some similarities between groups A and D when the Euclidean distance was 0.613. The concentrations of Lys/Met, Leu/Val, and Ala/Gly still maintained high consistency among the treatment groups. After 2 h of afternoon feeding, the amino acid patterns changed again among the treatments, with greater similarity observed between groups A and C, but significant differences remained compared to groups B and D. Among the treatments, the Ala/Gly, Leu/Val, and His/Phe continued to exhibited a high degree of similarity. After 4 h of afternoon feeding, the portal amino acid patterns of piglets in the four experimental treatment groups were divided into three clusters and groups A and B had the highest similarity. After 4h of afternoon feeding, the portal amino acid patterns in piglets from the four experimental treatment groups were divided into three cluster, but the groups A and B have the highest similarity. The amino acids with the highest similarity among the treatment groups were Pro/Glu, Leu/Val, Ala/Gly, Lys/Met, and Tyr/Ile.

### Relative expression of small intestinal mucosal glucose and amino acid transporter mRNAs

**Fig. 4:**
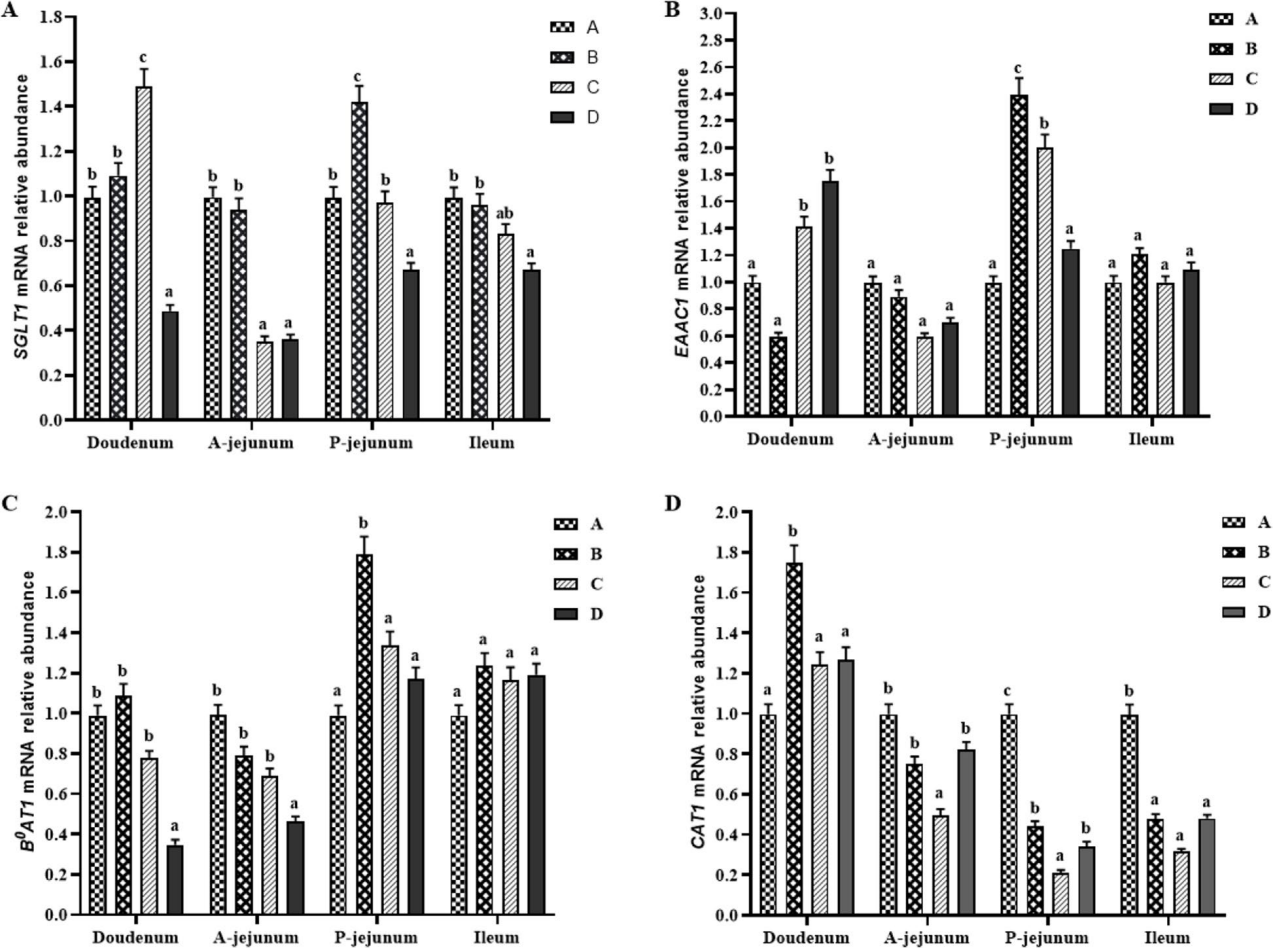
The relative expression of *SGLT1 *(**A**), *B*^*0*^*AT1 *(**B**), *EAAC1 *(**C**) and *CAT1 *(**D**) mRNA in individual segments of small intestine. ^a–c^Bars with different letters indicate statistically significant differences (*P* < 0.05)

As shown in Fig. 4, there was no significant difference between groups A and B in terms of the relative expression of the glucose transporter *SGLT1* gene in the duodenum of piglets, while group C had the highest expression (*P* < 0.05) and group D had the lowest (*P* < 0.05). In the intestinal epithelium of the anterior segment of the jejunum, groups A and B had similar expression and groups C and D had similar expression level of *SGLT1* mRNA; however, groups A and B had considerably higher expression levels than groups C and D (*P* < 0.05). In the posterior jejunum segment, groups A and C had considerably higher *SGLT1* gene expression than that in group D, while group B had significantly higher *SGLT1* gene expression than those in groups A and C (*P* < 0.05). However, in the ileum, only the mRNA level in group D was significantly lower than those in other groups. The relative mRNA expression of the amino acid transporter *B*^*0*^*AT1* was significantly higher in group D (*P* < 0.05) than in groups A, B, and C in the duodenum and anterior jejunum, although there was no significant difference between groups A, B, and C. In the posterior jejunum, only group B was significantly higher than the other groups (*P* < 0.05), while no difference between groups was shown in the ileum. Only the duodenum and posterior jejunum segments significantly differed in the relative expression of *EAAC1* mRNA between the groups (*P* < 0.05).

**Fig. 5:**
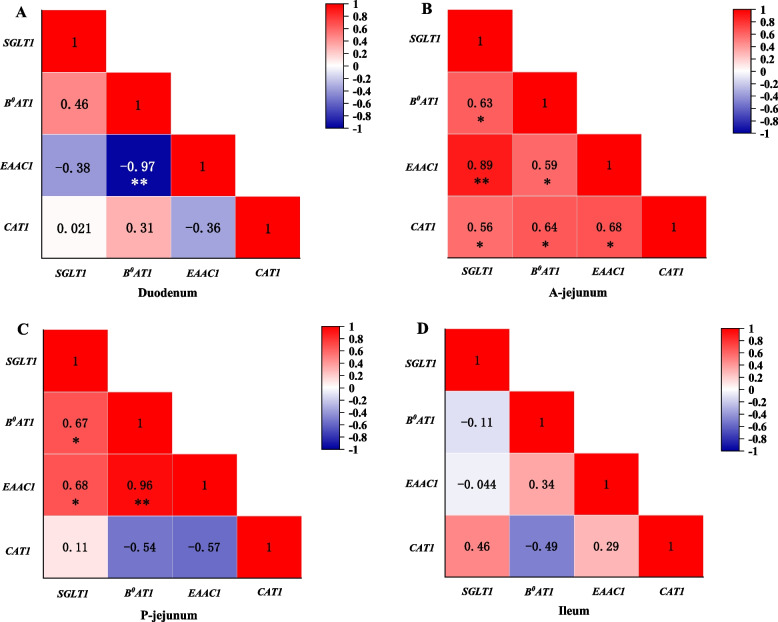
Correlation of mRNA expression levels of glucose and amino acid transporters in individual segments of small intestine. Heatmaps with correlation matrix depict Pearson’s correlation coefficients. Deep red and dark blue represent a stronger correlation coefficients and *P* values. Light red and light blue represent weaker correlation coefficients and *P* values. ^*^P < 0.05; ^**^P < 0.01

There were certain correlations between the mRNA expression of the glucose transporter *SGLT1* and the amino acid transporters *CAT1*, *B0AT1*, and *EAAC1* in the small intestine epithelium (Fig. 5). In the duodenal epithelium, except for the significant negative correlation between *B*^*0*^*AT1* and *EAAC1* mRNA expression (*P* < 0.05), the correlations between other transporters did not reach a significant level. However, the mRNA expression levels of all the transporters measured in the anterior jejunum epithelium significantly positively correlated (*P* < 0.05). In the posterior jejunum, *SGLT1* expression was significantly positively correlated with *B*^*0*^*AT1* and *EAAC1 *(*P* < 0.05), but *CAT1* expression was negatively correlated with *SGLT1*, *B*^*0*^*AT1*, and *EAAC1* to a certain extent (*P* > 0.05). In the ileum, there was still a correlation between the mRNA expression levels of the 4 transporters, but none of them reached statistical significance.

## Discussion

Swine diets are rich in carbohydrates, especially starch, which is the main energy source present in cereals, roots, tubers, and derivatives. The optimization of starch digestion is relevant to swine nutrition because it may increase the efficiency of energy metabolism and positively affect protein metabolism [15, 23]. Glucose is released from starch hydrolysis and is the main nutrient that influences glucose flux and insulin metabolism in the host, which in turn acts in the modulation of nutrient absorption and metabolism [12, 24–26]. Indeed, accumulating evidence indicates that diets containing starch with different amylose/amylopectin ratios distinctly alter growth performance and nitrogen utilization [25, 27, 28], and these changes may be associated with the alterations in the kinetics of diet glucose release [6, 29]. On the basis of our previous study on the effects of physicochemical and structural properties of single and double feedstuffs derived from different botanical sources on in vitro starch digestion and glucose release kinetics [30]. Four diets with different glucose release kinetics, containing similar levels of CP, NE, and starch, were formulated with starch sources from corn, corn/barley, corn/sorghum, or corn/cassava combinations in the present study. The results of in vitro digestion successfully verified the difference in glucose release kinetics among the 4 groups of experimental diets. The cassava, barley, maize, and sorghum exhibited distinct glucose release kinetics attributed to the variations in the content of rapidly digestible starch and the ratio of amylopectin to amylose. [30]. In this experiment, the corn/cassava diet exhibited the highest peak glucose release rate after 15 min of in vitro digestion, followed by the corn/barley, corn, and corn/sorghum diets. These findings are consistent with the glucose release patterns observed for cassava, barley, corn, and sorghum in previous study [30]. However, following the peak glucose release rate, the corn/cassava diet dramatically decrease the glucose release rate, while the corn/sorghum diet more gradually decreased the glucose release rate. Similarly, the corn/barley diet and corn diet were intermediate between the corn/cassava and corn/sorghum diet. Previous research has confirmed that the rate of in vitro starch digestion can roughly correspond to the rate of postprandial blood glucose change in growing pigs [6, 31]. Consequently, the present study investigated changes in portal glucose concentration over time following morning and afternoon feeding rather than investigating changes in blood glucose after meals in vivo. The portal glucose absorption kinetics in vivo and dietary glucose release kinetics in vitro were also consistent to some extent. For example, the glucose release rate of the group A diet was second only to that of the group B diet before 120 min of in vitro digestion, but the release rate was the fastest at 120 min, which was reflected in the portal glucose concentration after 2 h of morning feeding. The glucose release rate of the group B diet was higher than that of the other three groups at 30–90 min, and the cumulative glucose release was the highest, so the highest level was exceeded in the group A diet at 4 h after morning feeding. The changes in portal blood glucose levels in piglets after feeding indicate that the pattern of glucose release from the diet can affect the absorption of energy-carrier substances in the diet. This finding is consistent with that reported by Crapo et al. [32] and Deng et al. [33].

With the continuing relevant research on the optimization of dietary starch sources, scientists have gradually realized that glucose release kinetics and the dynamic balance of dietary glucose and amino acid supplies are the core factors through which dietary starch sources affect growth performance [18], protein digestion and metabolism [6] and energy utilization [29] in pigs. In this study, piglets fed corn/barley diet had lower fecal and urinary nitrogen excretion and the highest apparent protein digestibility throughout the trial. The small intestine is the primary site of nutrient absorption and that this process primarily relies on energy [34, 35]. As a result, glucose and amino acid oxidation are required to provide the necessary energy [36]. Diet B (corn/barley) can continuously and rapidly release glucose during 30–90 min of in vitro digestion, providing a continuous and stable energy supply for vital body functions such as nutrient absorption and transport and intestinal tissue metabolism. This diet may reduce amino acid oxidation in the intestine for energy supply and allow for absorption for tissue protein synthesis, ultimately enhancing nitrogen utilization. The greater amount of portal total free amino acids of piglets in group B also confirmed this inference. Conversely, piglets fed diet D (corn/cassava) exhibited higher fecal nitrogen excretion and significantly lower urinary nitrogen excretion compared to the traditional corn/soybean diet of groups A (*P* < 0.05). This finding suggests that the glucose release pattern of diet C and D has a negative effect on amino acid absorption in the small intestine but is advantageous for protein metabolism. The findings of the present study support the findings of Zhou et al. [6], who showed that piglets’ ability to efficiently utilize protein from their diet and deposit nitrogen can be enhanced by a diet that has a suitable glucose release kinetics pattern.

Glucose absorption in the intestine is necessary for the animals to reach full potential performance [37, 38]. It is also crucial for the intestinal absorption of other nutrients from the diet [39]. Previous studies have indicated that dietary starch sources significantly affect the net portal flux of amino acids and glucose in pigs [40, 41]. However, few studies evaluated the response of portal amino acid patterns to dietary glucose release kinetics. To further reveal the mechanism of the influence of dietary glucose release dynamics on nitrogen utilization, the present study used the Euclidean distance to perform cluster analysis of portal amino acid patterns and compared the differences in portal amino patterns of piglets receiving different diet treatments. The results demonstrated that after 2 h of morning feeding, the portal amino acid profiles of the piglets in all treatment groups exhibited significant disparities, subsequently diminishing gradually as the duration of feeding progressed. From the perspective of intergroup variation in amino acids, there was a consistent pattern observed among the groups for the Leu/Val and Ala/Gly from 2 h after morning feeding to 2 h after afternoon feeding, as did the Lys/Met at 2 h and 4 h after morning feeding. The levels of the aforementioned amino acids in the portal vein of piglets in group B were observed to be consistently relatively higher. The present findings suggest that the regulation of dietary glucose release kinetics can partially increase the portal concentrations of Gly, branched-chain amino acids Leu, Val and Ala, and the first and third limiting amino acids Lys and Met in piglets. However, the concentration patterns of Leu/Val, Ala/Gly and Lys/Met in portal vein blood remain relatively stable, showing less susceptibility to changes induced by dietary glucose release kinetics.

The potential role of amino acids as energy sources for mucosal cells is not new proposition. The catabolism and oxidation of enteral glutamate and aspartate by the intestinal tissues have been known for many years [42]. Glucose is also an important fuel for various tissues and cells, but previous studies have noted that under normal circumstances, glucose has difficulty inhibiting the oxidation of glutamate and glutamine, in contrast, it significantly reduces the oxidative metabolism rate of glucose [9, 43]. Indeed, the current study showed that dietary glucose release kinetics significantly affected portal Glu concentration in pigs at different times after feeding. Moreover, the portal Glu concentration was greater when the dietary glucose release and portal glucose levels were higher.

The small intestinal mucosa also plays an important role in degrading arginine, proline, and branched-chain amino acids and perhaps methionine, lysine, phenylalanine, threonine, glycine, and serine in the diet [10]. A large amount of ammonia is produced during the metabolism of these amino acids, and to prevent a large amount of ammonia from entering the liver and causing liver damage, some of the ammonia is converted into glycine and alanine which have lower molecular weight [44]. In addition, a previous study revealed that the proportions of different protein levels associated with the net absorption of glycine and alanine in the portal vein of piglets fed different diets were similar [44]. Leucine, isoleucine, and valine are collectively known as branched-chain amino acids. A previous study indicated that the first-pass metabolic rate of leucine and valine in piglet intestinal tissue is the same; both are 40%, while that of isoleucine is 30% [9, 41]. These findings are consistent with the findings of this study, which showed that the variations in glycine/alanine and Leu/Val between groups remained highly consistent at different times after feeding. Sodium–glucose cotransporters (SGLTs) constitute a large family of membrane proteins related to various modes of transport of glucose, amino acids, vitamins, and some ions across the apical membrane of the lumen side, including in the small intestine and renal tubule [45]. Intestinal *SGLT1* activity and expression are regulated by the dietary carbohydrate content. A previous study reported that *SGLT1* activity and expression increased in mice, rats, and sheep fed a high-sugar diet [46]. A high-glucose diet or a high-sodium diet reportedly increases the level of *SGLT1* expression in the small intestine [46, 47]. In this study, there was a certain level of consistency observed in the in vitro glucose release kinetics of the various diets, and the mRNA expression level of *SGLT1* in the intestinal epithelium and the portal glucose concentration after feeding showed a certain degree of consistency. For instance, after 15 min and 30 min of in vitro digestion, corn/cassava diet exhibited a higher glucose release rate than the other diets. Additionally, the expression of *SGLT1* mRNA in the duodenal epithelium was significantly higher in pig fed corn/cassava diet than in pigs fed the other diets. After 120 min of in vitro digestion, the glucose release rate of group A (corn diet) was higher than that of the other diets, followed by a higher portal glucose concentration 2 h after the morning feeding and a higher expression of *SGLT1* mRNA in the anterior jejunum. Certainly, the observed values at different time points during in vitro digestion may exhibit temporal and spatial discrepancies when compared to the results of in vivo experiments, which can potentially have an accumulative or delayed impact on glucose transporter expression in the intestinal epithelium as well as changes in portal glucose concentration. However, these findings can provide valuable insights and implications for future research.

To further understand the relationship between dietary glucose release dynamics and amino acid absorption, the correlation between the glucose transporter *SGLT1* and amino acid transporters was also analyzed. Although the entire small intestine is involved in digestion and absorption, most absorption of glucose and amino acids occurs in the jejunum, and some amino acid transporters, such as *B*^*0*^*AT1*, are usually expressed in lower amounts in the duodenum from the jejunum and increase steeply toward the ileum in pig [48]. For the duodenum and ileum, the present study revealed a statistically significant correlation only between the mRNA expression of the amino acid transporter *EAAC1* and that of *B*^*0*^*AT1* in the duodenal epithelium. A slower glucose release rate in the diet D not only downregulated the mRNA expression of the glucose transporter *SGLT1*, leading to a decrease in portal glucose concentration but also downregulated the mRNA expression of the amino acid transporter *B*^*0*^*AT1*; in contrast, the expression of *EAAC1* mRNA significantly increased. Excitatory amino acid carrier 1 (*EAAC1*) belongs to the EAAT family and is a Na^+^-dependent high-affinity Glu transporter. The duodenum is the starting place of digestion and absorption in the small intestine, and both the release and absorption of glucose are low. Especially in the case of excessively slow release of glucose from the D diet, the duodenum may need to absorb more Glu as an energy substrate for small intestine tissue, which may be the reason for the high expression of *EAAC1* mRNA. The transporter *B*^*0*^*AT1* accepts all neutral amino acids but prefers branched-chain amino acids and methionine [48]. The portal branched-chain amino acid concentrations, especially those of Leu and Ile of piglets in group D were consistent with the expression levels of *SGLT1* and *B*^*0*^*AT1* and were significantly lower than those in the other groups.

*SGLT1*, *B*^*0*^*AT1*, *EAAC1*, and *CAT1* expression levels in the jejunum epithelium were strongly correlated, especially in the anterior jejunum, and the expression levels above 4 transporters were significantly positively correlated. Overall, high expression of the glucose transporter *SGLT1* can promote the expression of the other three amino acid transporters and promote amino acid absorption. Since glucose transporters such as *SGLT1* and most amino acid transporters are Na^+^-dependent, some studies suggest that there may be competition between glucose and amino acid absorption and transport. A high-sodium diet reportedly increases the level of *SGLT1* expression in the small intestine [46, 47]. However, no negative correlation between *SGLT1* and amino acid transporters was found in the anterior jejunum segment in this study, which may also indicate that the glucose release level in the anterior jejunum segment in this study did not reach the level necessary for competition with amino acid transporters.

From the posterior jejunum to the ileum, the positive correlation between the expression of *SGLT1* and amino acid transporters gradually weakened. In the ileum, *SGLT1* expression even showed a certain degree of negative correlation with *B*^*0*^*AT1* and *EAAC1 *expression. This phenomenon may be related to the concentrations of glucose and amino acids in the ileum. Generally, starch is digested more rapidly than protein, especially in the proximal jejunum, and the ileal digestibility of starch is significantly higher than that of protein [1]. However, a study suggested that proteins releases amino acids faster than starch releases glucose in digestive tract of pigs fed low-protein diets [6]. Regardless of whether starch or protein is rapidly digested, the relative concentration of glucose in the ileum may be either higher or lower than that of amino acids. CATs are highly specialized for L-arginine, L-lysine, and L-ornithine transport, unlike their close homologs, namely, LAT transporters, which can also recognize neutral and anionic amino acids [49]. Compared with that in the other groups, the expression of *CAT1* in all the individual sections of the small intestine in group C was always the lowest, which was consistent with the findings of low portal appearance of Arg, Lysine, and His in the piglets in group C.

## Conclusion

Optimal dietary starch sources can effectively optimize the glucose release kinetic profile in the intestine of piglets, and this pattern may be the key factor through which dietary starch sources affect the growth performance and nutrient utilization of piglets. An appropriate dietary glucose release kinetic pattern can significantly improve dietary protein utilization in piglets. Thus, the proposed theory is that, initially, an optimized dietary glucose release kinetic pattern can enhance amino acid absorption and reduce fecal nitrogen excretion by upregulating the expression of amino acid transporters in the intestinal epithelium. On this basis, suitable kinetic patterns of dietary glucose release can improve the portal amino acid profile, effectively strengthen protein deposition, and reduce urinary nitrogen excretion in piglets. In the present study, corn/barley diet produced a more appropriate kinetics pattern of glucose release.

## Data Availability

The data analyzed during the current study are available from the corresponding author upon reasonable request.

## Abbreviations

Ala: Alanine
Arg: Arginine
Glu: Glutamicacid
Gly: Glycine
His: Histidine
Ile: Isoleucine
Leu: Leucine
Lys: Lysine
Met: Methionine
PDV: Portal-drained viscera
Phe: Phenylalanine
Pro: Proline
Thr: Threonine
Tyr: Tyrosine
Val: Valine
